# Primary Outcome from a cluster-randomized trial of three formats for delivering Community Reinforcement and Family Training (CRAFT) to the significant others of problem drinkers

**DOI:** 10.1186/s12889-022-13293-8

**Published:** 2022-05-10

**Authors:** Rikke Hellum, Randi Bilberg, Kjeld Andersen, Gallus Bischof, Morten Hesse, Anette Søgaard Nielsen

**Affiliations:** 1 The Unit of Clinical Alcohol Research (UCAR), Institute of Clinical Research, University of Southern Denmark, J.B. Winsløws vej 18, 5000 Odense C, Denmark; 2 Psychiatric Department, Region of Southern Denmark, Odense, Denmark; 3 The Department of Psychiatry and Psychotherapy, University of Luebeck, Ratzeburger Allee 160, 23562 Lübeck, Germany; 4 Centre for Alcohol and Drug Research, Department of Psychological and Behavioral Sciences, Aarhus University, Artillerivej 90, 2, 2300 Copenhagen S, Denmark

**Keywords:** Community Reinforcement and Family Training, CRAFT, Concerned significant others, Alcohol treatment, Treatment engagement, Alcohol use disorder

## Abstract

**Background:**

Community Reinforcement and Family Training (CRAFT) is an intervention designed to help the concerned significant others (CSOs) of people with alcohol problems who are reluctant to seek treatment. It aims to improve the well-being of CSOs and teach them how to change their behavior in order to positively influence the “identified patient” (IP) to seek treatment.

**Methods:**

The aim of the present pragmatic cluster-randomized trial was to compare the effectiveness of three formats for delivering CRAFT in real life settings: group sessions, individual sessions, and written material only (control group). Eighteen public treatment centers for alcohol use disorders were randomly assigned to deliver CRAFT in one of the three formats as part of their daily clinical routine. CSOs were recruited via pamphlets, general practitioners, and advertisements on social media. Trained clinicians delivered CRAFT in individual and group format, and self-administered CRAFT was limited to handing out a self-help book. The primary outcome was treatment engagement of the IP after three months.

**Results:**

A total of 249 CSOs were found to be eligible and randomly assigned to receive CRAFT delivered in group, individual, or self-administered format. The three-month follow-up rate was 60%. At three months follow-up, 29% (*n* = 32) of the CSOs who received group/individual CRAFT reported that their IP had engaged in treatment. The corresponding rate for the CSOs who received self-administered CRAFT was lower (15%; *n* = 5) but did not differ significantly from the other group of CSOs (Odds ratio (OR) = 2.27 (95% CI: 0.80, 6.41)).

**Conclusion:**

We hypothesized that CSOs receiving CRAFT in a group format would improve the most, but although our findings pointed in this direction, the differences were not statistically significant.

**Trial registration:**

Clinical trials.gov ID: NCT03281057. Registration date:13/09/2017.

**Supplementary Information:**

The online version contains supplementary material available at 10.1186/s12889-022-13293-8.

## Introduction

Alcohol use disorder (AUD) causes serious consequences for the persons who suffer from the condition, but lately there has been an increased focus on those who have a close relationship with the drinker, the so-called concerned significant others (CSOs). The CSOs often live in stressful circumstances and have lots of worries that frequently lead to poorer physical and mental health [1] as well as lower quality of life [2] compared to the general population. Moreover, this group of CSOs often experience loneliness and stigmatization [3].

In Denmark, 140,000 people are assumed to suffer from alcohol dependence [4]; however, only around 17,000 Danes are enrolled in specialized treatment each year [5]. Moreover, it is often the case that the person with AUD has been suffering from the disorder for 10 years or more before entering treatment [6]. As a result, both persons with AUD and their CSOs may, at length, suffer from the consequences of alcohol use.

Interventions for CSOs, independent from treatment for the individual with the substance use disorder (i.e., ‘identified patient’; IP), have been developed. To date, the approach with the best evidence is Community Reinforcement and Family Training (CRAFT), a behavioral intervention that aside from improving the well-being of CSOs, teaches them how to change their behavior in order to positively influence the IP to seek treatment. A meta-analysis of 11 studies on CRAFT delivered in different formats, involving CSOs of persons suffering from alcohol and/or drug misuse or gambling, found that CRAFT was twice as effective in engaging the IPs to treatment compared to control/comparison groups [7]. Studies on problematic gambling found the lowest IP treatment entry rates, all below 25%. Also, the IP treatment engagement rates varied according to the type of format used to deliver CRAFT to the CSOs. In studies where the CSOs were offered individual therapy, 12.5–71% of the IPs subsequently engaged in treatment, and in two studies in which the CSOs were offered support in a group format, 60% of the IPs engaged in treatment. Interventions aimed at the CSOs consisting of a mix of individual and group sessions have, so far, been found to lead to the highest IP treatment engagement rates: 77–86% [7]. Group format is considered a cost-effective way of providing CRAFT, and a study by Manuel and colleagues indicated that CRAFT delivered in closed group format may be just as effective as individual CRAFT, but the sample size was indeed small and the study did not compare the two formats directly [8]. Whether CRAFT delivered in open group format is as effective as individual CRAFT has yet to be investigated. Open and closed groups have different advantages and disadvantages. Closed group formats are often preferred since they allow the same group of participants to meet and get to know one another well and to go through a logical chain of topics from start to finish. However, closed group formats often involve waiting lists, since it is not possible for new participants to join the intervention until a new group starts. In contrast, an open group format allows for new participants to join an existing group and blend in with the other participants. The order of the topics in the open group is similar to that of closed formats, but, consequently, this means that some new group members may, so to speak, start in what may be considered to be the middle of a chain of topics and continue until they reach the middle of the chain again. The advantage of using open group formats is, thus, that they are easier to implement (it is quicker to fill up a group) and easier to access (no waiting list), but they may require more work on the therapist’s part in order to repeatedly explain to new participants where the group is in the chain of topics and how the topics relate to one another. We expect to find higher improvement in the quality of life of the CSOs receiving group CRAFT than those receiving individual CRAFT, since the CSOs in group CRAFT may benefit from the dynamics that occur in a group of individuals sharing, at least in part, similar circumstances. Moreover, being part of a group may create a sense of mutual recognition and may lower the feeling of isolation and shame among CSOs [9].

One study has tested CRAFT as a self-administered intervention and the results indicate that this format has a similar, albeit less pronounced, effect to individual and group formats, with a treatment engagement rate of 40% [8].

Although CRAFT is one of the most studied methods aimed at helping CSOs to motivate IPs to enter treatment for AUD, only very few studies have tested CRAFT in a European context [10–12]. Most previous studies have tested the impact of CRAFT on IP treatment entry rates and only few studies have reported other measures such as improvement in the mental health of CSOs and impact on family cohesion [10, 12].

In sum, the most studied format for delivering CRAFT is individual counseling, and only a few studies have analyzed CRAFT delivered in group format. There is some evidence that CRAFT based on self-help materials leads to elevated rates of IP treatment entry. Most studies, so far, took place in a research-based environment [8, 12]. So, although there are several high-quality randomized control trials, the effectiveness of different CRAFT-formats under regular treatment conditions is still unclear. The present study was thus designed as a pragmatic trial operating within real-life conditions. The study followed the implementation of CRAFT interventions into the daily routine of Danish community-based alcohol treatment centers, and the therapists involved were staff from the alcohol treatment centers participating in the study. The aim of the present study was to investigate whether one of the three formats for delivering CRAFT (individual, open group, self-administered) is more effective than the others in getting problem drinkers to seek treatment for their alcohol problems, and whether one format has a larger impact on the quality of life of the CSOs than the others. When planning the study, we hypothesized that:

1. CSOs randomly assigned to receive six sessions of CRAFT delivered in either individual format or group format with a continuous enrollment of CSOs would be able to motivate their IP to enter treatment significantly more often than CSOs randomly assigned to a control condition consisting of self-administered CRAFT.

2. Six sessions of CRAFT delivered in open group format with continuous enrollment of CSOs would improve the quality of life and psychological functioning of the CSOs significantly more than both individual and self-administered CRAFT.

The present study is not only the first to examine group CRAFT in Europe, but it is also the first to investigate CRAFT delivered in an open group format [9]. 

## Methods

### Design

The 18 treatment centers comprised both larger institutions with more than 25 staff members and small centers with fewer staff members. The treatment centers were randomized to deliver CRAFT to the CSOs in one of the following three formats:

1. CRAFT as six individual sessions with a therapist, supported by written material.
2. CRAFT as six open group sessions, supported by written material. The groups started when two CSOs had contacted the treatment facility and continuously included new members. Each CSO followed six group sessions with one or two therapists.
3. Control condition, consisting of CRAFT delivered in a self-administered format and by means of written material only.

### Randomization

The treatment centers (*n* = 18) were randomized to deliver CRAFT in individual, open group, or self-administered format. Thus, each facility was assigned to deliver CRAFT in one of the three formats to all CSOs who approached the facility for support during the study period. CSOs were eligible to participate in the study if they approached the treatment facility expressing concern for their IP’s drinking habits and were not already in treatment or had received treatment in the past three months. Due to low capacity, it was assumed that the small centers could not recruit enough CSOs to run a group within a reasonable time frame. Therefore, we chose to perform a cluster randomization in three stages. The three large centers were the first to be randomized to deliver CRAFT in one of the three formats, followed by the smallest centers, and then the remaining medium-sized centers. The randomization was performed in the computer program STATA, by giving the participating treatment centers random numbers and then randomly assigning them to one of the three conditions. The randomizations were blinded and performed by an independent person not involved in the study. The CSOs were not told beforehand which intervention each facility had been allocated to. The participating centers were spread out over Denmark.

Consecutive CSOs who contacted a center that had been randomized to deliver either individual or group CRAFT were offered the particular intervention within two weeks of an intake interview. Both individual and group CRAFT consisted of six sessions with 7–10 days between each session [9]. CSOs in all three groups began with an intake interview where they were interviewed, asked about potential violence and threats from the drinkers, and filled out the baseline questionnaire. In the case of risk of violence being present, the CSO was given advice on how to receive specific help.

Consecutive CSOs who contacted a center that had been randomized to deliver self-administered CRAFT were offered an intake interview and, afterwards, written material only. At the intake interview, the CSOs were informed that they could have an individual follow-up session with a therapist after three months for additional support, if needed. This individual follow-up session was offered after the primary outcome had been measured and it was added to ensure that the CSOs in the control group felt that they had received adequate help. The self-administered intervention was chosen as a control condition instead of either ‘treatment as usual’ or a waiting list. Treatment as usual was disregarded since the usual interventions being offered to the CSOs differ between the treatment centers. Some centers offer brief advice over the phone, while other centers offer group-based psychoeducation or individual personal support delivered face to face. A waiting list was disregarded as a control condition since participants waiting for an intervention or treatment do not act ‘naturally’, but simply wait and become worse than they would have outside the study [13], potentially leading to an overestimation of the effectiveness of the experimental condition. Instead, we decided to offer the control group written material with the possibility of a follow-up face-to-face session with a therapist after three months and regarded this to be an appropriate minimal intervention.

### Participants

#### Recruitment

To disseminate the information on CRAFT interventions being available to the public and the possibility of CSOs needing to seek it, information leaflets and posters were distributed by the participating local authorities. The local authorities were committed to distributing the leaflets via social services departments, departments for children and adolescents, and general practitioners and others who might come into contact with CSOs. The title of the leaflet was “Alcohol—are you concerned about someone who drinks too much? – Help is available…” The leaflet emphasized that it is hard to be close to someone with alcohol problems. CRAFT was introduced as being of help to the significant other, but the type of delivery format used by the local alcohol treatment institutions was not described. The leaflet also provided information on where to receive a CRAFT intervention, i.e., the address and telephone number of the local alcohol treatment institution. Additionally, the alcohol institutions used advertisements in local newspapers and videos and posts on social media, linked to the alcohol treatment centers’ websites and their Facebook pages. Further, information about the project was posted on national websites for counseling on alcohol problems such as Alkohol & Samfund (in English: “Alcohol & Society”) and the National Telephone Hotline ‘Alkolinjen’ [14].

#### Inclusion criteria (CSO)

Any individual with a close relationship to someone with AUD could participate in the trial if they met the following criteria: 1) 18 years or older; 2) being a CSO with concern for an IP’s drinking habits; 3) not currently receiving treatment for an alcohol problem; 4) have the intention of maintaining contact with the center for the next 90 days; 5) have had regular contact with the IP for the past 90 days (face-to-face contact for several hours on, at least, a weekly basis) or the desire to re-establish regular contact with an IP; and 6) being prepared, at least to some extent, to support the IP if they should choose to seek treatment.

#### Exclusion criteria (CSO)

CSOs were excluded if they 1) suffered from dementia or other cognitive disorders; 2) did not speak Danish; 3) were psychotic or otherwise severely mentally ill; 4) had been receiving treatment for alcohol problems for the past three months; and 5) were concerned about a person who, according to the CSO, mainly used illegal substances.

The therapist who enrolled the CSO estimated whether he/she fulfilled the inclusion criteria and screened for exclusion criteria. All CSOs who sought help through one of the participating treatment centers and fulfilled the criteria were offered the CRAFT format that the facility had been randomized to. No other interventions aimed at helping CSOs were offered at the participating treatment centers during the study period.

### Questionnaires

After enrollment and before the first session, the CSOs completed a self-administered questionnaire (baseline, t0) on a tablet, starting with an informed consent form. Data were collected again after three months (t1) and six months (t2) by a self-administered Web-based battery of questionnaires or by telephone interview. The participants received up to three reminders for the follow-up questionnaire until they had responded. Data on whether and when IPs started treatment were collected from the CSOs three and six months after enrollment of the CSOs into the study.

### Measures and variables

#### Demographics

Demographic information (only at baseline) included gender, age, and level of education.

#### Additional information on the CSO

We asked the CSOs whether they 1) worked full time (yes or no); 2) had been on sick leave within the past 30 days (yes or no); 3) had children living at home (yes or no); and 4) had previously sought counselling because of their IP’s alcohol use (yes, no, or don’t know). Further, the Alcohol Use Disorder Identification Test (AUDIT) [15] was used to collect information on the CSOs’ use of alcohol. Response options for each question were coded 0–4 and summed. The scores were classified into three groups: < 8, 8–15, and > 15.

#### Information on the IP

We asked the CSOs to provide the age and gender of their IP and to indicate the type of relationship they had with them (partner, daughter/son, parent, or other). We also asked the CSOs how often they had spent time with their IP in the past 4 weeks (almost every day, 5 times a week, 3 times a week, 2 times a week, or 2 times a month), how often the IP drinks (daily, mainly on weekdays, mainly on weekends, usually one day per week, usually less than one day per week, or don’t know), and whether the IP had been in treatment for AUD before (yes, no, or don’t know).

### Outcome measures

#### Primary outcome

The primary outcome was the proportion of IPs who entered alcohol treatment between baseline and three months after enrollment of their CSO to the study. The primary outcome was assessed by asking the CSOs whether their IP had entered treatment during this period (no, yes, or don’t know). CSOs who responded “don’t know” were excluded from the analysis. A total of 7 (5%) and 8 (6%) CSOs were excluded after three and six months, respectively. If there was a missing response at both 3 and 6 months the variable was missing.

#### Secondary outcomes

1. Changes in depression symptoms of the CSOs following the CRAFT intervention
2. Changes in the quality of life of the CSOs

To assess changes in the quality of life of the CSOs following the CRAFT intervention, we used the four subscales of the World Health Organization Quality of Life instrument (WHOQOL): Physical Health, Psychological, Social Relationships, and Environment. All four subscales are scored from 1 to 5, with a higher score indicating a higher level of quality of life [16]. The Patient Health Questionnaire-9 (PHQ-9) was used to assess changes in depression symptoms of the CSOs following the CRAFT intervention (scores 0–4 = no depression symptoms; scores 5–27 = depression symptoms) [17].

### Intervention

#### Therapist training and supervision

The recruitment of the CSOs and the interventions were conducted between January 1^st^, 2018, and December 31^st^, 2019. The therapists taking part in the study were regular employees at the treatment centers and comprised social workers, nurses, and psychologists, most of them with special training in Motivational Interviewing and all of them experienced in working with patients with AUD and their relatives. The therapists who delivered individual or group CRAFT underwent a three-day course in CRAFT before delivering the intervention. The therapists delivering the control intervention were not trained until after conclusion of the study period to avoid spill-over effects. The training was undertaken by one of the authors (GB) in charge of a German study of CRAFT during the years 2008–2009 [12]. The training consisted of an examination of all the elements in CRAFT, including practicing and role-play. In addition, the therapists received brush-up training after six months. The therapists were guided by a treatment protocol and each session was documented. The treatment sessions followed a protocol, based on the CRAFT manual [18], and all sessions were audio recorded in preparation to ensure treatment adherence and supervision of the therapists.

To ensure fidelity to the CRAFT method and therapist style, two authors (ASN and MH) listened to a randomly drawn sample of the audiotapes recorded during treatment sessions. Feedback was given to the therapists, as well as feedback on additional specific sessions, if the therapist asked for this. The therapists received feedback on a minimum of two recordings of their sessions if they delivered individual CRAFT or co-performed in a facility randomized to deliver group CRAFT. Only three therapists asked for feedback on a specific session. The project group met with key therapists from each treatment center each month during the first year of the project and every second month during the last year of the project. Here, a current status of the project was given, cases were discussed, and additional supervision was given.

### Interventions for CSOs

#### Individual and group sessions

Previous CRAFT studies conducted with the CSOs of persons with AUD mostly offered 12–14 sessions of CRAFT to the CSOs [7]. In the present study, the number of sessions for individual CRAFT was reduced to six sessions of one hour, and for group CRAFT the number of sessions was reduced to six two-hour sessions. The reason for this was two-fold: partly because if the interventions prove successful, an intervention consisting of six sessions is assumed to have a fair chance to be implemented in the daily routine of clinical practice in a Danish context, free of charge for the CSOs; and partly because the findings of previous CRAFT studies suggest that IP treatment engagement is typically realized within the first six sessions [19].

Both the individual and group CRAFT interventions covered the following eight topics [18]: 1) Strengthening the motivation of the CSO; 2) Training in recognition of early signs of domestic violence, particularly as new behavioral change techniques are introduced, intentionally designed to be experienced as negative by the IP; development of a safety plan; 3) Training in functional analyses to outline the triggers of the drinking problem as well as the positive and negative consequences of it; 4) Training in identifying the CSO’s own unintentional role in the maintenance of the IP’s using cycle; training in effective communication with the IP; 5) Training in appropriate and consistent use of positive reinforcement of the IP’s non-using prosocial (non-drinking) behavior; 6) Training in positive reinforcement; learning to reinforce clean and sober behavior by using small rewards; 7) Training the withdrawal of reinforcement at times of drinking episodes to allow for the natural negative consequences of the IP’s using behavior; help to identify the CSO’s own areas of life dissatisfaction and training the development of specific plans for addressing that dissatisfaction and in rewarding themselves more often; 8) Training the methods on how and when to suggest treatment to an IP, including the development of a “rapid intake” plan and working with how to handle disappointments in a fruitful way.

#### Self-administered CRAFT format (control)

CSOs randomized to the control condition only received written material and were considered to be controls for the first three months after enrollment. The written material was a brief, easy-to-read book [20], inspired by the American CRAFT self-help book “Get Your Loved One Sober” [21], and in particular by the German written support material”Strategien zur Selbsthilfe für Angehörige von Menschen mit Alkoholproblemen, Der Community Reinforcement Ansatz: das Familien-Training (CRAFT)” [22], which was used in the German study on CRAFT [12]. The book described the eight topics covered in CRAFT including violence. In addition, the book included a chapter containing basic information about the mechanisms in AUDs, and how AUD affects both IPs and CSOs, as well as information about alcohol treatment, what treatment implies, and how to easily get access to treatment. All three groups received the book: the control-group received the book as the only intervention, and participants in the individual and group conditions received the book as an additional support to the face-to-face interventions.

### Treatment for IP

In Denmark, treatment for AUD does not require a referral and it is free of charge to all citizens. By law, treatment has to be offered within two weeks, and individuals may choose to seek treatment at their local treatment center or at a treatment center in another municipality if they prefer. In contrast to previous studies, no special treatment for the IPs was integrated within the CRAFT interventions in the present study. However, if an IP became motivated to seek treatment, free treatment was immediately made available at the treatment center where the CSO had received the CRAFT intervention, or in any other community-based treatment center. The CSOs were thoroughly informed about treatment options for IPs, e.g., free specialized alcohol treatment and treatment in general practice.

### Statistical analysis

Data were analyzed using Stata version 16.1. The primary outcome was compared between the three CRAFT groups at three- and six-months follow-up using logistic regression. To check for imbalance in the baseline values between the three randomized groups, we used analysis of variance (ANOVA) [23]. A combined group was created comprising the CSOs who received individual or group CRAFT, and the primary outcome was compared between this combined group and the self-help CRAFT group at both follow-up times. Corresponding analyses were conducted on a combination of the primary outcome at three- and six-months follow-up: IPs who were engaged in treatment at *either* three- or six-months follow-up were compared to IPs who were not engaged in therapy at any time. Secondary outcomes were compared between the three CRAFT groups at three- and six-months follow-up, using linear regression with robust standard errors and analysis of covariance, adjusting for baseline values of the outcomes, with robust standard errors. We did not adjust for other covariates since there were too few answers/participants in some categories. Further, another combined group was created comprising the CSOs who received individual or self-help CRAFT, and the secondary variables were compared between this combined group and the group that received CRAFT in group format. All pairwise comparisons of the primary and secondary outcomes between the three CRAFT groups were adjusted for multiple testing using Sidak’s correction [24]. The three- and six-months analyses were based on Intention-to-treat. Characteristics of the participants and dropouts at both three- and six-months follow-up were compared using chi-square tests for categorical covariates, and Student’s unpaired *t*-tests for numerical covariates. The analyses were checked by a third person. The datasets used and/or analyzed during the current study are available from the corresponding author upon reasonable request.

A power calculation was based on an expected 60% of the CSOs receiving either group or individual CRAFT, and 40% of the CSOs receiving written material, to be able to motivate the IP to enter treatment, based on data from Manuel and colleagues [8]. Effects of CRAFT were tested one-sided since previous studies consistently demonstrated improvement in all outcome measures in individuals receiving CRAFT [18]. Based on these expectations, 106 participants in each group were needed to be able to detect a 20-percentage point difference in the primary outcome between the CRAFT individual/group intervention and the CRAFT self-help intervention, with an α level at 5%, and a power of 90%. As we predicted a dropout rate of approximately 10% and, additionally, a lost to follow-up rate of 10%, we needed to include 131 CSOs in either group.

### Ethics approval and consent to participate

This study was approved by the Danish Data Protection Agency (Region of Southern Denmark 2008–58-0035 project no. 17/46074). The study was submitted for ethical approval to the Danish Ethics Committee (Project-ID: S-20170148) but we were informed that the study did not require formal approval since it was a questionnaire survey to compare different ways of implementing a recommended treatment method, CRAFT, according to the National Clinical Guidelines in Denmark.

All participants were informed, both orally and in writing, about the procedures for attending the study. The participants signed an informed consent document prior to participating in the study. All relevant guidelines have been followed according to the Declaration of Helsinki.

The study was registered at ClinicalTrials.gov Identifier: NCT03281057.

## Results

### Study sample

**Fig. 1:**
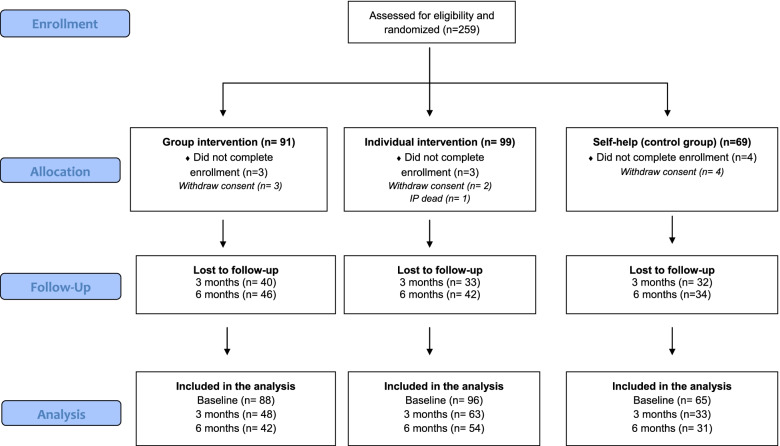
Design of the study and flow of the participants

During the study period from 2018 – 2019, a total of 259 CSOs (see Fig. 1) were included in the study. After filling out the baseline questionnaire, 10 CSOs dropped out of the study or withdrew their consent to participate, leaving 249 participants: *n* = 88 in the group CRAFT intervention, *n* = 96 in the individual CRAFT intervention, and *n* = 65 in the self-help (control) intervention. Among the 249 participants in the study, 60% (*n* = 151) completed the three months follow-up assessment: 56% (*n* = 49) in the group CRAFT intervention, 70% (*n* = 67) in the individual CRAFT intervention, and 57% (*n* = 37) in the control intervention. At six months follow-up, 55% (*n* = 136) of all the participants completed the questionnaire: 50% (*n* = 44) in the group CRAFT intervention, 59% (*n* = 57) in the individual CRAFT intervention, and 54% (*n* = 35) in the control intervention. A dropout analysis (see supplementary table 2) was made for those who did not complete the three- or six- months follow-up. The analysis showed that the ones who did not answer at three- or six-months follow-up were younger and scored lower on the Quality-of-Life domain environment (DOM4) at baseline.

### Sample

Baseline characteristics of the CSOs and IPs are presented in Table 1. The CSOs were mostly female (85% *n* = 211), and the mean age of the participants was 49 years (SD; 13.9). The most common relation to the IP was partner/spouse (50% *n* = 123), the second most common was daughter/son (22% *n* = 53), and the third most common was parent (12% *n* = 28). More than half of the CSOs (60%; *n* = 148) had children living at home, and 22,5% (*n* = 56) of the CSOs had previously sought counseling because of their IP’s drinking. The gender of the IPs was mostly male (73% *n* = 182), their mean age was 55 years (SD; 13.3), and 40% (*n* = 99) of the IPs had previously been in treatment for their drinking. CSOs who reported that the IP was drinking mainly on a daily basis comprised 56% (*n* = 138) of the sample, and 15% (*n* = 37) of the participants reported that the IP was mainly drinking during weekends, and 6% (*n* = 15) reported that the IP was drinking less than once a week. At baseline, we found missing responses for level of education (*n* = 2), work (*n* = 2), sick leave (*n* = 2), children (*n* = 2), AUDIT (*n* = 5), relation to IP (*n* = 3), sex of IP (*n* = 1), IP drinking pattern (*n* = 4), and IP earlier counselling (*n* = 4). In accordance with GDPR (General Data Protection Regulation) less than 5 participants in one response category was listed as < 5.

**Table 1:** Characteristics of study population, stratified by intervention group

	Total study sample	CRAFT intervention		
		Group	Individual	Self-help
**Number of participants, n**	249	88	96	65
**Sex, n (%)**				
Male	38 (15)	9 (10)	16 (17)	13 (20)
Female	211 (85)	79 (90)	80 (83)	52 (80)
**Age, mean (SD)**	49.0 (13.9)	47.3 (14.6)	51.6 (13.7)	47.7 (12.9)
**Level of education, n (%)**				
Less than university degree	208 (84)	75 (86)	80 (84)	53 (82)
University degree	39 (16)	12 (14)	15 (16)	12 (18)
**Work full or part time, n (%)**				
No	86 (35)	37 (43)	31 (33)	18 (28)
Yes	161 (65)	50 (57)	64 (67)	47 (72)
**Have been on sick leave within the last 30 days**				
No	211 (85)	71 (82)	84 (88)	56 (86)
Yes	36 (15)	16 (18)	11 (12)	9 (14)
**Children living at home, n (%)**				
No	148 (60)	61 (70)	54 (57)	33 (51)
Yes	99 (40)	26 (30)	41 (43)	32 (49)
**Earlier counselling, n (%)**				
Yes	56 (22.5)	23 (26)	22 (23)	11 (17)
No	191 (76.7)	65 (74)	74 (77)	52 (80)
Do not know	< 5^a^	0	0	< 5^a^
**AUDIT score, n (%)**				
< 8	206 (84)	66 (77)	83 (88)	57 (89)
8–15	36 (15)	19 (22)	10 (11)	7 (11)
> 15	< 5^a^	< 5^a^	< 5^a^	0
**Relationship to the IP, n (%)**				
Partner/spouse	123 (50)	42 (48)	55 (59)	26 (41)
Daughter/son	28 (11)	12 (14)	8 (9)	8 (13)
Parent	53 (22)	18 (20)	18 (19)	17 (27)
Other	42 (17)	16 (18)	13 (14)	13 (20)
**Sex of the IP, n (%)**				
Male	182 (73)	69 (79)	70 (73)	43 (66)
Female	66 (27)	18 (21)	26 (27)	22 (34)
**Age of the IP, mean (SD)**	55.3 (13.3)	54.0 (12.9)	56.4 (13.3)	55.3 (13.8)
**How often have you spend time with the IP in the last 4 weeks, n (%)**				
Almost Everyday	133 (53)	43 (49)	59 (61)	31 (48)
5 times a week	13 (5)	8 (9)	< 5^a^	< 5^a^
3 times a week	20 (8)	8 (9)	5 (5)	7 (11)
2 times a week	29 (12)	10 (11)	12 (13)	7 (11)
2 times a month	44 (18)	16 (18)	14 (15)	14 (22)
Not within the last 4 weeks	10 (4)	< 5^a^	< 5^a^	5 (8)
**How does the IP drink at the moment (according to the CSO), n (%)**				
Daily	138 (56)	49 (56)	51 (54)	38 (59)
Mainly on the weekdays	12 (5)	< 5^a^	7 (7)	< 5^a^
Mainly on the weekends	37(15)	15 (17)	12 (13)	10 (16)
Usually one day per week	11 (4)	< 5^a^	5 (5)	< 5^a^
Usually less than one day per week	15 (6)	5 (6)	7 (7)	< 5^a^
Do not know	32 (13)	12 (14)	12 (13)	8 (13)
**Has the IP earlier been in treatment, n (%)**				
No	135 (55)	50 (57)	47 (51)	38 (58)
Yes	99 (40)	35 (40)	40 (43)	24 (37)
Do not know	11 (4)	< 5^a^	6 (6)	< 5^a^
**Quality of life at baseline, mean (SD)**				
DOM1 Physical Health^b^	15.1 (2.6)	15.1 (2.6)	14.9 (2.6)	15.2 (2.9)
DOM2 Psychological^b^	12.9 (2.7)	12.8 (2.6)	13.0 (2.5)	13.1 (3.1)
DOM3 Social Relationships^b^	13.0 (2.9)	12.9 (2.8)	12.9 (2.9)	13.4 (2.9)
DOM4 Environment^b^	14.4 (2.0)	14.7 (2.1)	14.3 (1.8)	14.4 (2.0)
**Depression at baseline, mean (SD)**				
PHQ-9^c^	8.2 (5.4)	8.7 (5.6)	7.9 (4.9)	8.0 (5.8)

Missing observations: Level of education (*n* = 2), work (*n* = 2), sick leave (*n* = 2), children (*n* = 2), audit (*n* = 5), relation to IP (*n* = 3), sex of IP (*n* = 1), IP drinking pattern (*n* = 4), IP earlier counselling (*n* = 4)

^a^Less than 5 participants, precise number omitted due to GDPR

^b^WHOQOL Measuring Quality of Life

^c^Patient Health Questionnaire (PHQ-9)

The WHOQol-score at baseline was highest for the domain Physical Health (DOM1), with an average score of 15.1 (SD 2.6). The score for the domain Psychological (DOM2) was 12.9 (SD 2.7) on average. The domain Social Relationships (DOM3) was 13.0 (SD 2.9) on average, and the score for the domain Environment was 14.4 (SD 2.0) on average.

The average PHQ-9 (depression) score for all participants was 8.2 (SD 5.4). The score was highest for participants in the group CRAFT intervention (8.7 (SD 5.6)) and lowest for participants in the individual CRAFT intervention (7.9 (4.9)).

### Intervention outcomes

#### Primary outcome: treatment engagement

Table 2 shows the percentage of IPs who had entered treatment three months following the enrollment of their CSO to the study. At three months follow-up, 29% (*n* = 14) of the CSOs who received group CRAFT, and 29% (*n* = 18) of the CSOs who received individual CRAFT, reported that their IP had engaged in treatment. In the control group, 15% (*n* = 5) of the CSOs engaged their IP to treatment. The difference between the intervention for individual/group vs. control group was not statistically significant (Odds ratio (OR) = 2.27 (95% CI: 0.80, 6.41)). An additional analysis (see supplementary table 1) showed an IP treatment engagement rate of 43% after six months among the CSOs who received individual or group CRAFT, and the corresponding rate in the control group was 32%. There was no significant difference between the intervention for individual/group vs. control group (Odds ratio (OR) = 1.61 (0.66, 3.97)).

**Table 2:** Treatment engagement at three- and six months’ follow-up, comparisons of CRAFT intervention groups

	Total study sample^a^	CRAFT intervention				Pairwise comparisons of CRAFT intervention groups			
		Group	Individual	Self-help	*Group or individual*	Group vs. Individual	Group vs. self-help	Individual vs. self-help	*Group or individual vs. self-help*
	N (%)	N (%)	N (%)	N (%)	*N (%)*	OR (95%-CI)	OR (95%-CI)	OR (95%-CI)	*OR (95%-CI)*
N	144	48	63	33	111				
**Treatment engagement at 3 months**									
No	107 (74)	34 (71)	45 (71)	28 (85)	*79 (71)*	Ref	Ref	Ref	*Ref*
Yes	37 (26)	14 (29)	18 (29)	5 (15)	*32 (29)*	1.03 (0.37, 2.83)	2.31 (0.57, 9.26)	2.24 (0.59, 8.57)	*2.27 (0.80, 6.41)*
**Treatment engagement at 6 months**		42	54	31	96				
N	127	42	54	31	96				
No	94 (74)	30 (71)	38 (70)	26 (84)	*68 (71)*	Ref	Ref	Ref	*Ref*
Yes	33 (26)	12 (29)	16 (30)	5 (16)	*28 (29)*	0.95 (0.32, 2.82)	2.08 (0.50, 8.68)	2.19 (0.56, 8.62)	*2.14 (0.74, 6.16)*

^a^Excluding participants who answered “Don’t know” to question on the IP’s treatment engagement at three months’ follow-up: At 3 months *n* = 7 (5%); at six months *n* = 8 (6%)

#### Secondary outcomes

##### Quality of life score for the CSOs

The change in the WHOQol-score among the CSOs from baseline to three months follow-up is presented in Table 3. The CSOs who received group CRAFT reported an increase in the WHOQol-score from baseline to three months follow-up in all four quality of life domains except for the environment domain (-0.36, SD: 1.67). The CSOs who received individual CRAFT reported an increase in scores in all four domains. The CSOs in the control group reported an increase in scores in the physical health domain (DOM1) (0.56, SD: 2.03) and the psychological domain (DOM2) (0.04, SD: 1.80), and a decrease in scores in the domains of social relationships (DOM3) (-0.22 SD: 2.15) and environment (DOM4) (-0.47 SD:1.49). There were no significant differences between the group intervention vs. individual/control intervention (DOM1: adjusted mean difference: -0.12 [95% CI: -0.74, 0.50]; DOM2: adjusted mean difference: 0.25 [95% CI: -0.46, 0.96]; DOM3: adjusted mean difference: 0.68 [95% CI: -0.07, 1.43]; DOM 4: adjusted mean difference = -0.15 [95% CI: -0.76, 0.47]).

**Table 3:** Change in psychological functioning from baseline to three months’ follow-up, comparisons of CRAFT intervention groups

	Total study sample	CRAFT intervention				Pairwise comparisons of CRAFT intervention groups							
		Group	Individual	Self-help	*Individual or self-help*	Group vs. Individual		Group vs. self-help		Individual vs. self-help		*Group vs. individual or self-help*	
	Mean change (SD)	Mean change (SD)	Mean change (SD)	Mean change (SD)	*Mean change(SD)*	Mean change diff. (95%-CI)		Mean change diff. (95%-CI)		Mean change diff. (95%-CI)		*Mean change diff. (95%-CI)*	
**Quality of life**						Un-adjusted	Adjusted	Un-adjusted	Adjusted	Un-adjusted	Adjusted	*Un-adjusted*	*Adjusted*
DOM1 Physical Health^b^	0.72 (1.94)	0.61 (1.85)	0.89 (1.97)	0.56 (2.03)	*0.78 (1.99)*	-0.28 (-1.16, 0.59)	-0.27 (-1.10, 0.56)	0.05 (-0.98, 1.09)	-0.16 (-0.81, 0.49)	-0.12 (-0.74, 0.50)	0.41 (-0.50, 1.32)	*-0.16 (-0.81, 0.49)*	*-0.12 (-0.74, 0.50)*
DOM2 Psychological^b^	0.59 (2.09)	0.72 (2.13)	0.80 (2.19)	0.04 (1.80)	*0.53 (2.08)*	-0.08 (-1.07, 0.91)	-0.05 (-1.00, 0.90)	0.68 (-0.35, 1.71)	0.20 (-0.53, 0.92)	0.25 (-0.46, 0.96)	0.83 (-0.11, 1.79)	*0.20 (-0.53, 0.92)*	*0.25 (-0.46, 0.96)*
DOM3 Social Relationships^b^	0.37 (2.23)	0.86 (2.42)	0.32 (2.07)	-0.22 (2.15)	*0.13 (2.10)*	0.53 (-0.51, 1.58)	0.52 (-0.56, 1.50)	1.08 (-0.12, 2.28)	0.73 (-0.07, 1.53)	0.68 (-0.07, 1.43)	0.47 (-0.55, 1.48)	*0.73 (-0.07, 1.53)*	*0.68 (-0.07, 1.43)*
DOM4 Environment^b^	-0.09 (1.73)	-0.36 (1.67)	0.34 (1.83)	-0.47 (1.49)	*0.05 (1.75)*	-0.70 (-1.50, 0.10)	-0.45 (-1.26, 0.36)	0.11 (-0–72, 0.94)	-0.41 (-0.99, 0.17)	-0.15 (-0.76, 0.47)	**0.87 (0.14, 1.59)**	*-0.41 (-0.99, 0.17)*	*-0.15 (-0.76, 0.47)*
**Depression**													
PHQ-9^c^	-1.94 (4.91)	-2.02 (6.52)	-1.97 (3.32)	-1.78 (4.15)	*-1.90 (3.93)*	-0.05 (-2.61, 2.51)	0.43 (-1.71, 2.57)	-0.24 (-3.06, 2.58)	-0.12 (-2.14, 1.89)	0.21 (-1.46, 1.87)	-0.61 (-2.39, 1.16)	*-0.12 (-2.14, 1.89)*	*0.21 (-1.46, 1.87)*

^a^Adjusted for baseline value of outcome

^b^WHOQOL Measuring Quality of Life

^b^Questionnaire about depression (PHQ-9 Danish)

##### Depression score for the CSOs

The change in the PHQ-9 depression score from baseline to three months follow-up is also presented in Table 3. All three groups showed a decrease in the depression score at three months follow-up, with a mean change for all CSOs at -1.94 (SD:4.91). The decrease was highest among the CSOs who received group CRAFT (-2.02, SD: 6.52), followed by the CSOs who received individual CRAFT (-1.97, SD: 3.32), and, lastly, the CSOs in the control group (-1.78, SD: 4.15). However, the differences between the groups were not significant (adjusted mean difference = 0.21 [95% CI: -1.46, 1.87]).

## Discussion

This study of CRAFT is the largest to date (*n* = 249). It is the first study to investigate three formats for delivering CRAFT in the same study, one being the format of open groups, as well as the first study to be performed as an effectiveness trial in real-life settings and with a relatively low number of exclusion criteria. While the results favored the group and individual formats over the self-help format after both three- and six-months follow-up, the difference between the groups was not statistically significant.

In the present study, the IP treatment engagement rate was 29% after three months, which is lower than what has been reported in previous studies of CSOs of persons with substance use disorders [7]. Although treatment initiation of IPs typically occurs within the first three months after enrollment of the CSO in a CRAFT intervention, additional IPs enroll in treatment during the period 3–6 months after enrollment of the CSO [12, 25]. In the present study, the IP treatment engagement rate after six months increased to 43%. Previous studies on CRAFT for CSOs of individuals with alcohol and/or drug problems reported slightly higher rates of IP treatment entry six months following enrollment of the CSO in the CRAFT intervention, i.e., 48–64% [8, 12, 19, 26, 27]. After six months, there only seems to be a very small increase in treatment engagement rate from 6 to 12 months [12, 19].

In the present study, we did not find a significant difference in the IP treatment engagement rate between the interventions. Of the CSOs who received open-group CRAFT, 49% had engaged their IP in treatment at six months follow-up. So far, only one other study has investigated the impact of delivering CRAFT in group format to CSOs of people with alcohol and/or drug problems [8]. The study reported a treatment engagement rate of 60% at six-months follow-up for CSOs who intended to engage in the study, and the rate for all the CSOs who were enrolled in the study was 71%. In contrast to the present study, Manuel and colleagues investigated the impact of a closed group format [8]. Since closed group formats without continuous enrollment of new group members tend to imply waiting lists for the new participants, we investigated the impact of an open group format with continuous enrollment of new CSOs. The outcome of such open groups, measured as treatment engagement rate of the IP, did not differ significantly from individual sessions, but we learned that it was manageable to deliver CRAFT in such a format.

In this study, the IPs of 39% of the CSOs who received individual-CRAFT had sought treatment at six months follow-up of the CSOs. Previous studies on CRAFT delivered as individual sessions reported treatment engagement rates of 48–64% after six months [11, 19, 27, 28]. One explanation for the lower treatment engagement rate for both group and individual CRAFT in the present study compared to prior studies might be because we reduced the number of CRAFT sessions to six instead of the 10–14 sessions offered in previous studies [8, 12, 19, 27, 28], and that the CSOs, therefore, did not have the time needed to train, for instance, communication skills. In the study on iCRAFT, i.e., CRAFT delivered in an online format, the authors investigated the impact of five online sessions [10], and similar to our study, they also found a lower treatment engagement rate at six months than did prior studies, namely 21.6%. The reasons for investigating the impact of a lower number of CRAFT sessions were several. First, we considered it more manageable for the CSOs to join a shortened intervention. Second, we considered it easier and more likely, in the long run, to implement a shorter CRAFT format in the public treatment institutions, compared to an intervention consisting of twice as many sessions. Moreover, a previous study indicated that the treatment engagement for the IP already took place after 4–6 CRAFT sessions with the CSO [19], and we therefore considered that six sessions might be a reasonable treatment intervention. However, it should be considered that reducing the number of sessions from 10–12 to six might not leave enough time for the CSOs to get rid of their frustrations. Orford and colleagues have criticized CRAFT for being too focused on motivating the IP to seek treatment and not offering unconditional support to the CSO [29]. When shortening the CRAFT intervention, the time for unconditional support for the CSO may even be less, and this should, of course, be taken into consideration when planning to implement a CRAFT intervention in daily clinical routine. In most previous CRAFT studies, the potential treatment of the IP was not integrated into the CRAFT intervention or particularly more accessible due to the CRAFT intervention. Another and perhaps more plausible explanation for the lower treatment rate might thus be that in Denmark, the public treatment of individuals with alcohol problems and support for their CSOs are already easily accessible to both groups and is offered free of costs for the individual, since it is financed by the municipalities. This has been the situation for CSOs the last 20 years at least, and for IPs even longer. Since the treatment and support for both IPs and the CSOs are generally easily accessible in Denmark, the intervention in the present study may not necessarily lead to a high level of increased treatment engagement for the IPs than by offering the interventions in a culture where treatment participation is costly for the individual. The study of the impact of the intervention involved access to treatment free of charge for the IP [8, 19, 30].

Finally, the reason for the relatively low rate of treatment initiation among the IPs may be due to our inclusion criteria being broader than other studies as far as who could be considered as a CSO, and less strict regarding the amount of time CSOs had to spend with their IPs, regardless of the type of relationship. Like other studies on CRAFT [7], most of our study participants were female (85%). The percentage of spouses/partners composed 50%, parents 22%, and daughters/sons composed 11%. Thus, our spouse/partner percentage was lower than all other CRAFT studies (alcohol) [7]. In some studies, with a high treatment engagement rate, it was an inclusion criterion that the CSO and IP were living together or beings relative, spouse, intimate partner [19, 31]. Therefore, the lower proportion of spouses/partners might also have influenced the present study’s results since this group of CSOs can be considered to spend more time with their IP and have a larger capacity to influence their IP. However, our broader inclusion criteria mirror the real-life situation, where both friends, siblings, and ex-partners approach the alcohol treatment institutions for help for their loved ones.

Our three months follow-up rate at 60% was lower than we had hoped for. An analysis of the CSOs, lost-to-follow-up at three- and six months follow-up, showed that those who were lost to follow-up were significantly younger than those who were not. Furthermore, the CSOs lost-to-follow-up scored lower on the quality-of-life domain “Environment” when included in the study. In this study, the follow-up questionnaires were sent to the participants by post, personal email, or completed with an interviewer during a phone call, depending on the wishes expressed by the CSOs at the time of enrollment in the study. Despite several reminders being sent out, follow-up by post or via secure email may have influenced the low rate of follow-up since people, particularly younger people, tend to forget to check the secure email account or forget to post mail. In another study in which the follow-up was done via personal interviews, the research group managed to achieve a follow-up rate of 100% at three- and six-months follow-up and 92% at 12-months follow-up [12]. Another study paid participants for their participation in the follow-up interviews and, thus, also achieved high follow-up rates (70–90%) [8].

It was not only a challenge to receive a sufficiently high number of follow-up data, but it also turned out to be an overall challenge to inform CSOs about the possibility of receiving help and seeking professional help, i.e., participating in the CRAFT-study. There may be many reasons for this. Firstly, the alcohol treatment centers participating in this study experienced that most of the CSOs who approached them for support and help had IPs that were already seeking treatment. Therefore, these CSOs were excluded from participation in the present study and, instead, involved directly in the treatment of the IP. Secondly, CSOs, in general, find it challenging to seek professional help [32]. On the one hand, they are convinced that they can solve the problem themselves [33], and some CSOs are in serious doubt about whether there is a problem or not [29]. Thus, the CSOs often wait quite a long time before seeking help and they are often worn out when they reach this point [29]. The challenge to recruit CSOs might also be due to treatment for AUD being considered taboo, and a common barrier is the lack of knowledge about treatment [34]. Drinking alcohol is a social norm in Denmark and something which almost everyone does [35]. Alcohol is associated with pleasure and quality of life [36, 37] but when the drinking gets too much, it is seen as a taboo, stigmatized and hard to address, even among persons working in the health care system [38].

In the present study, the amount of support received by the CSOs in CRAFT individual or group format. CRAFT might also vary, since the CSOs were included in the analysis independently of whether they participated in only a few or all the six sessions of the intervention (intention to treat approach). Therefore, some of the CSOs may have received relatively little help and might, therefore, not have acquired the skills to increase the chance of engaging the IP to treatment.

Earlier studies on CRAFT have used a waiting list, treatment as usual or another type of CSO-intervention as a control group [8, 12, 19]. Since the support for CSOs in Denmark is very diverse throughout the country, ‘treatment as usual’ was not considered an option for a control group in the present study. Instead, we opted for the control group to receive one single session with a therapist introducing the study and offering self-administered CRAFT, based on written material (a book), to serve as a minimal intervention. For ethical inferior reasons it was stressed that the CSO would be welcome to return and receive further face-to-face support if, after three months, the CSOs felt that self-administered CRAFT was insufficient. In the control group, 32% of the CSOs ended up motivating their CSO to treatment after six months, which indicates that a third of this group was helped through a minimum of intervention. Thus, in several aspects, the present study's control intervention is rather similar to the iCRAFT study intervention [10], where the intervention consisted of five online sessions with videos, text to read, exercises, and homework assisted by a therapist. In this study, the control group was a waiting list, offered to intervention after 24 weeks, and 21,3% engaged their IP to treatment after six months [10]. It indicates that even with a relatively low-level intervention, some CSOs can engage their IP in treatment. The relatively high engagement rate for the control group in this study might also be why it was not possible to show a statistically significant difference between the intervention groups and the control group, compared to other studies using a waiting list. This might give preference to the experimental treatment because people randomized to a waiting list seem to wait their turn for the treatment. Thus, they do not work on changing their behavior or engaging their IP in treatment to the same extent as they would if they had not participated in a study [39]. This is in line with Manuel et al., who also found a numerical, but not statistical, difference between CSOs who received the self-help material and those who received the in-person intervention. The secondary outcomes of the present study were quality-of-life and mental health. In most domains, the CSOs increased their quality-of-life, and CSOs from all three intervention groups recorded decreased depression scores after three months follow-up. As we hypothesized, the CSOs from the group intervention had the highest decrease, although this difference was not statistically significant. During and following participation in CRAFT, the CSOs experienced a reduction in depression scores. These findings are consistent with earlier studies' findings [10, 12].

Effect sizes of scientific trials are difficult to maintain in real-life settings [40], and this was also the case in the present study. In the future, we suggest investigating offering a mixture of individual and group CRAFT sessions to CSOs, since this has been found to be most effective [7]. Based on our findings from a qualitative sub-study on CRAFT participation, we would also suggest offering all CSOs a follow-up session to keep their minds on the intervention [41]. It might also be of interest to compare the outcomes of CRAFT to the outcomes of other theoretical models like, e.g., 5-step-method [42].

### Strengths and limitations

Some limitations of the present study are to be mentioned. It is an important limitation that despite the high number of the participants, the present study is still underpowered, especially since a substantial number of participants could not be reached at follow-up and, thus, had to be excluded from the analysis. The number of lost-to-follow-up is relatively high, and it may not be possible to generalize our findings, particularly regarding younger CSOs. One massive challenge was to recruit enough CSOs to the study, which contributed to the fact that we did not reach the number of participants considered in the power calculation. We had a hard time reaching the CSOs and making them aware of this new intervention. Even though we tried to call attention to the study through the distribution of flyers, videos, and posters in public places and on social media, we should have used even more resources to publish in the media from the beginning. We did not find it possible, with the time available, to enroll the number of CSOs, that we aimed for.

In line with previous studies on CRAFT, data on treatment entry of the IP was collected from the CSO only, and that may also be considered a limitation. Because of the Danish data restrictions, we were not allowed to collect data on the person with an alcohol use disorder from the treatment centers. Therefore, there may be IPs who entered treatment without telling their CSOs, and this may have led to a lower treatment entry than reported in the study. Furthermore, it must also be considered a limitation that we do not know the exact number of CSOs from the self-administered CRAFT who made use of an individual follow-up session after three months of enrollment in the study. Also, data on the ‘number of attended sessions’ were not collected consistently. However, it is our impression that most CSOs participated in most of the sessions. This impression is based on the reporting from the therapists involved.

A further limitation is that we did not assess level of adherence and excluded clinicians who fell short of a pre-defined standard. Thus, the quality of services delivered in both the group and individual setting may have varied considerably. The assessment of the quality of CRAFT delivered was monitored by listening to randomly chosen recordings of the sessions and overall, the quality was considered good. This might, however, not be the case for sessions not picked for control. Further, no validated evaluation tool was used for the assessment. Three therapists specifically asked for feedback and did that on a specific session. All other feedback was given on sessions randomly chosen to assess the intervention’s fidelity.

The strength of the study is, that it was a pragmatic trial operating within real-life conditions, which is important for future implementation in daily clinical practice. Moreover, it is the largest study of CRAFT so far, with 249 CSOs, and the first study to compare CRAFT in three formats.

## Conclusion

Overall, to increase the likelihood that the IP seeks treatment, the present study did not demonstrate a robust advantage of offering CRAFT in open group format or as individual counselling over offering self-help materials alone. Still, this finding should be received with caution due to the potential lack of power in the study. We hypothesized that the CSOs receiving CRAFT in a group format would themselves improve the most, but although our findings pointed in this direction, the differences were not statistically significant. 

## Supplementary Information

**Additional file 1.**

**Additional file 2.**

## Data Availability

The datasets used and/or analyzed during the current study are available from the corresponding author on reasonable request.

## Abbreviations

AUD: Alcohol use disorders
CRAFT: Community Reinforcement and Family Training
CSO: Concerned significant other
IP: Identified Patient

## References

[1] Greenfield TK, Karriker-Jaffe KJ, Kerr WC, Ye Y, Kaplan LM (2016). Those harmed by others' drinking in the US population are more depressed and distressed. Drug Alcohol Rev.

[2] Birkeland B, Foster K, Selbekk AS, Hoie MM, Ruud T, Weimand B (2018). The quality of life when a partner has substance use problems: a scoping review. Health Qual Life Outcomes.

[3] Di Sarno M, De Candia V, Rancati F, Madeddu F, Calati R, Di Pierro R (2020). Mental and physical health in family members of substance users: a scoping review. Drug Alcohol Depend.

[4] Hansen AB, Hvidtfeldt UA, Gronbaek M, Becker U, Nielsen AS, Tolstrup JS (2011). The number of persons with alcohol problems in the Danish population. Scand J Public Health.

[5] Danish Health Authority. Sundhedsstyrelsens servicetjek af offentligt finansieret alkoholbehandling: Sundhedsstyrelsen; 2019. Available from: https://www.sst.dk/-/media/Udgivelser/2020/Alkoholservicetjekket/Sundhedsstyrelsens-servicetjek-paa-offentligt-finansieret-alkoholbehandling.ashx?la=da&hash=.

[6] Schwarz AS, Nielsen B, Nielsen AS (2018). Changes in profile of patients seeking alcohol treatment and treatment outcomes following policy changes. Zeitschrift fur Gesundheitswissenschaften. J Public Health.

[7] Archer M, Harwood H, Stevelink S, Rafferty L, Greenberg N (2020). Community reinforcement and family training and rates of treatment entry: a systematic review. Addiction (Abingdon, England).

[8] Manuel JK, Austin JL, Miller WR, McCrady BS, Tonigan JS, Meyers RJ (2012). Community reinforcement and family training: a pilot comparison of group and self-directed delivery. J Subst Abuse Treat.

[9] Hellum R, Nielsen AS, Bischof G, Andersen K, Hesse M, Ekstrom CT (2019). Community reinforcement and family training (CRAFT) - design of a cluster randomized controlled trial comparing individual, group and self-help interventions. BMC Public Health.

[10] EÉk N, Romberg K, Siljeholm O, Johansson M, Andreasson S, Lundgren T (2020). Efficacy of an internet-based community reinforcement and family training program to increase treatment engagement for AUD and to improve psychiatric health for CSOs: a randomized controlled trial. Alcohol Alcohol.

[11] Bisetto Pons D, González Barrón R, Botella GÁ (2016). Family-Based Intervention Program for parents of substance-abusing youth and adolescents. J Addict.

[12] Bischof G, Iwen J, Freyer-Adam J, Rumpf HJ (2016). Efficacy of the Community Reinforcement and Family Training for concerned significant others of treatment-refusing individuals with alcohol dependence: a randomized controlled trial. Drug Alcohol Depend.

[13] Redko C, Rapp RC, Carlson RG (2006). Waiting time as a barrier to treatment entry: perceptions of substance users. J Drug Issues.

[14] Alkohol & Samfund. https://alkohologsamfund.dk/: https://alkohologsamfund.dk/;

[15] Babor TF, Higgins-Biddle JC, Saunders JB, Monteiro MG, Dependence WHODoMHaS. AUDIT : the Alcohol Use Disorders Identification Test : guidelines for use in primary health care. Geneva: World Health Organization; 2001.

[16] The World Health Organization (1995). The World Health Organization quality of life assessment (WHOQOL): position paper from the World Health Organization. Soc Sci Med.

[17] Kroenke K, Spitzer RL, Williams JB (2001). The PHQ-9: validity of a brief depression severity measure. J Gen Intern Med.

[18] Smith JEMR (2004). Motivating substance abusers to enter treatment : working with family members.

[19] Miller WR, Meyers RJ, Tonigan JS (1999). Engaging the unmotivated in treatment for alcohol problems: a comparison of three strategies for intervention through family members. J Consult Clin Psychol.

[20] Nielsen AS. Selvhjælpsbog til pårørende til mennesker med alkoholproblemer- Hjælp til at få din pårørende i behandling- og hjælp til dig selv, så du får større glæde i dit liv. Odense: Unit of Clinical Alcohol Research; 2017.

[21] Meyers RJ WB. Get your loved one sober. Alternatives to nagging, pleading, and threatening. Hazelden Center City, Minnesota. 2004.

[22] Brück R (2008). Strategien zur Selbsthilfe für Angehörige von Menschen mit Alkoholproblemen.

[23] Gliner JA, Morgan GA, Harmon RJ (2003). Pretest-posttest comparison group designs: analysis and interpretation. J Am Acad Child Adolesc Psychiatry.

[24] Šidák Z (1967). Rectangular confidence regions for the means of multivariate normal distributions. J Am Stat Assoc.

[25] Sisson RW, Azrin NH (1986). Family-member involvement to initiate and promote treatment of problem drinkers. J Behav Ther Exp Psychiatry.

[26] Kirby KC, Benishek LA, Kerwin ME, Dugosh KL, Carpenedo CM, Bresani E (2017). Analyzing components of community reinforcement and family training (CRAFT): is treatment entry training sufficient?. Psychol Addict Behav.

[27] Dutcher LW, Anderson R, Moore M, Luna-Anderson C, Meyers RJ, Delaney HD (2009). Community Reinforcement and Family Training (CRAFT): an effectiveness study. J Behav Anal Health Sports Fit Med.

[28] Kirby KC, Benishek LA, Kerwin ME, Dugosh KL, Carpenedo CM, Bresani E (2017). Analyzing components of Community Reinforcement and Family Training (CRAFT): is treatment entry training sufficient?. Psychol Addict Behav.

[29] Orford J, Velleman R, Natera G, Templeton L, Copello A (1982). Addiction in the family is a major but neglected contributor to the global burden of adult ill-health. Soc Sci Med.

[30] Kirby KC, Marlowe DB, Festinger DS, Garvey KA, La MV (1999). Community reinforcement training for family and significant others of drug abusers: a unilateral intervention to increase treatment entry of drug users. Drug Alcohol Depend.

[31] Meyers RJ, Miller WR, Smith JE, Tonigan JS (2002). A randomized trial of two methods for engaging treatment-refusing drug users through concerned significant others. J Consult Clin Psychol.

[32] McCann TV, Lubman DI (2018). Help-seeking barriers and facilitators for affected family members of a relative with alcohol and other drug misuse: a qualitative study. J Subst Abuse Treat.

[33] Hellum R, Bilberg R, Nielsen AS. “He is lovely and awful”: The challenges of being close to an individual with alcohol problems. Nordic Studies on Alcohol and Drugs. 2022;39(1):145507252110448. 10.1177/14550725211044861 PMC8899274 35308468

[34] May C, Nielsen A, Bilberg R. Barriers to treatment for alcohol dependence. J Drug Alcohol Res. 2022;39(1):8.

[35] Grønkjær M, Curtis T, De Crespigny C, Delmar C (2011). Acceptance and expectance: Cultural norms for alcohol use in Denmark. Int J Qual Stud Health Well Being.

[36] Emiliussen J, Andersen K, Nielsen AS (2017). Why do some older adults start drinking excessively late in life? Results from an Interpretative Phenomenological Study. Scand J Caring Sci.

[37] Elmeland K. Alkohol- og ruskultur i Danmark. Mennesker med alkoholproblemer : baggrund, belastning, behandling. 1. udgave ed. Kbh.: Nyt Nordisk Forlag i samarbejde med Dansk Sygeplejeråd; 2015. p. 34–44.

[38] Hellum R, Bjerregaard L, Nielsen AS (2016). Factors influencing whether nurses talk to somatic patients about their alcohol consumption. Nordic Stud Alcohol Drugs.

[39] Miller WR (2015). No more waiting lists. Subst Use Misuse.

[40] Blonde L, Khunti K, Harris SB, Meizinger C, Skolnik NS (2018). Interpretation and impact of real-world clinical data for the practicing clinician. Adv Ther.

[41] Hellum R, Bilberg R, Bischof G, Nielsen AS (2021). How concerned significant others experience Community Reinforcement and Family Training (CRAFT) – a qualitative study. BMC Fam Pract.

[42] Copello A, Templeton L, Orford J, Velleman R (2010). The 5-Step Method: evidence of gains for affected family members. Drugs: Educ Prev Policy.

