# Supplementary material for: Primary Outcome from a cluster-randomized trial of three formats for delivering Community Reinforcement and Family Training (CRAFT) to the significant others of problem drinkers
Source: BMC Public Health. 2022 May 10;22:928. doi: 10.1186/s12889-022-13293-8 (PMC9087923; doi:10.1186/s12889-022-13293-8)
Supplement: Supplementary file 1 — Additional file 1. [file 12889_2022_13293_MOESM1_ESM.docx]

## Supplementary Table 1: Treatment engagement at either three- or six-months’ follow-up^a^, pairwise comparisons of CRAFT intervention groups

|  | Total study  sample | CRAFT intervention | | | | Pairwise comparisons of CRAFT intervention groups | | | |
| --- | --- | --- | --- | --- | --- | --- | --- | --- | --- |
|  |  | Group | Individual | Self-help | *Group or individual* | Group vs. Individual | Group vs. self-help | Individual vs. self-help | *Group or individual vs. self-help* |
|  | N (%) | N (%) | N (%) | N (%) | *N (%)* | OR (95%-CI) | OR (95%-CI) | OR (95%-CI) | *OR (95%-CI)* |
|  | 118 | 41 | 49 | 28 | 90 |  |  |  |  |
| **Treatment engagement^b^** |  |  |  |  |  |  |  |  |  |
| No | 70 (59) | 21 (51) | 30 (61) | 19 (68) | *51 (57)* | Ref. | Ref. | Ref. | *Ref.* |
| Yes | 48 (41) | 20 (49) | 19 (39) | 9 (32) | *39 (43)* | 1.50 (0.5, 4.20) | 2.01 (0.59, 6.85) | 1.33 (0.40, 4.43) | *1.61 (0.66, 3.97)* |

^a^Participants with treatment engagement at either 3 or 6 months against patients with no treatment engagement at both 3 or 6 months. The “don’t know” category comprises participants replying at both time points AND replying “Don’t know” at either 3 or six months AND not replying “Yes” at either time point.

^b^Excluding participants in the “Don’t know” category, n=9 (7%)
