# Supplementary material for: Primary Outcome from a cluster-randomized trial of three formats for delivering Community Reinforcement and Family Training (CRAFT) to the significant others of problem drinkers
Source: BMC Public Health. 2022 May 10;22:928. doi: 10.1186/s12889-022-13293-8 (PMC9087923; doi:10.1186/s12889-022-13293-8)
Supplement: Supplementary file 2 — Additional file 2. [file 12889_2022_13293_MOESM2_ESM.docx]

## Supplementary Table 2: Drop out analyses at 3 and 6 months

|  | Total study sample | Three months follow-up | | | Six months follow-up | | |
| --- | --- | --- | --- | --- | --- | --- | --- |
|  |  | Participating | Drop out | *p*-value | Participating | Drop out | *p*-value |
| **Number of participants, n** | 249 | 151 | 98 |  | 136 | 113 |  |
| **Sex, n (%)** |  |  |  | 0.272 |  |  | 0.789 |
| Male | 38 (15) | 20 (13) | 18 (18) |  | 20 (15) | 18 (16) |  |
| Female | 211 (85) | 131 (87) | 80 (82) |  | 116 (85) | 95 (84) |  |
| **Age, mean (SD)** | 49.0 (13.9) | 51.2 (12.7) | 45.7 (15.0) | **0.002** | 51.3 (12.6) | 46.2 (14.9) | **0.004** |
| **Relation to the IP, n (%)** |  |  |  | 0.373 |  |  | 0.934 |
| Partner/spouse | 123 (50) | 79 (53) | 44 (45) |  | 68 (51) | 55 (49) |  |
| Daughter/son | 28 (11) | 16 (11) | 12 (12) |  | 15 (11) | 13 (12) |  |
| Parent | 53 (22) | 27 (18) | 26 (27) |  | 27 (20) | 26 (23) |  |
| Other | 42 (17) | 27 (18) | 15 (15) |  | 24 (18) | 18 (16) |  |
| **IP earlier counselling, n (%)** |  |  |  | 0.187 |  |  | 0.748 |
| No | 135 (55) | 83 (55) | 52 (55) |  | 72 (53) | 63 (57) |  |
| Yes | 99 (40) | 64 (42) | 35 (37) |  | 56 (41) | 43 (39) |  |
| Do not know | 11 (4) | <5^a^ | 7 (7) |  | 7 (5) | <5^a^ |  |
| **Quality of life at baseline, mean (SD)** |  |  |  |  |  |  |  |
| DOM1 Physical Health^b^ | 15.1 (2.6) | 15.1 (2.8) | 15.0 (2.4) | 0.823 | 14.9 (2.8) | 15.2 (2.4) | 0.397 |
| DOM2 Psychological^b^ | 12.9 (2.7) | 13.0 (2.8) | 12.9 (2.5) | 0.885 | 12.9 (2.8) | 13.0 (2.5) | 0.813 |
| DOM3 Social Relationships^b^ | 13.0 (2.9) | 13.0 (2.9) | 13.1 (2.8) | 0.745 | 13.0 (3.0) | 13.1 (2.8) | 0.878 |
| DOM4 Environment^b^ | 14.4 (2.0) | 14.7 (2.0) | 14.0 (1.9) | **0.012** | 14.7 (2.0) | 14.1 (1.8) | **0.021** |
| **Depression at baseline, mean (SD)** |  |  |  |  |  |  |  |
| PHQ-9^c^ | 8.2 (5.4) | 8.3 (5.8) | 8.1 (4.6) | 0.831 | 8.1 (5.6) | 8.4 (5.0) | 0.763 |

^a^Less than 5 participants, precise number omitted due to GDPR

^b^WHOQOL Measuring Quality of Life.

^c^Questionnaire about depression (PHQ-9 Danish)
